# Evaluation of a Lung Nodule in the Setting of Metastatic Gastrointestinal Stromal Tumor: Surgical Considerations in Carney’s Triad

**DOI:** 10.7759/cureus.7620

**Published:** 2020-04-10

**Authors:** Dathe Benissan-Messan, Emily Singer, Peter Kneuertz, Robert Merritt, Desmond D'Souza

**Affiliations:** 1 Gastrointestinal and General Surgery, The Ohio State University Wexner Medical Center, Columbus, USA; 2 Thoracic Surgery, The Ohio State University Wexner Medical Center, Columbus, USA; 3 Surgery, The Ohio State University Wexner Medical Center, Columbus, USA

**Keywords:** cancer, chest, genetic syndromes, lung, benign or congenital lesions, metastases/metastasectomy

## Abstract

In 1977, Carney et al. first described an association of gastric epithelioid leiomyosarcoma or gastrointestinal stromal tumor (GIST), pulmonary chondroma, and extra-adrenal paraganglioma. This previously unrecognized disorder came to be known as Carney’s triad. We describe a case of a 27-year-old female with metastatic GIST, diagnosed with Carney’s triad following pulmonary wedge resection, and highlight the surgical implication of this rare disease association.

## Introduction

Carney triad is a rare syndrome characterized by the association of gastrointestinal stromal tumor (GIST), paraganglioma, and pulmonary chondroma. The etiology of the disorder is unknown. Diagnosis relies on a high index of suspicion from the clinician and management is surgical. 

## Case presentation

A 27-year-old female presented with a history of abdominal pain. CT imaging of the abdomen revealed multiple gastric masses, hepatic lesions, bilateral adrenal adenomas, and a well-circumscribed pulmonary mass in the right lower lobe. Positron emission tomography revealed hypermetabolic activity in stomach, adrenal gland, retroperitoneal nodes, and in the right lung. CT-guided percutaneous lymph node biopsy of the gastric mass revealed a GIST (<5 mitoses/50 HPF, CD117 positive, negative for CD34, smooth muscle actin [SMA], cytokeratin, S100. Ki-67 4%). The patient was diagnosed with metastatic GIST and started on a highly selective tyrosine kinase inhibitor. 

**Figure 1:**
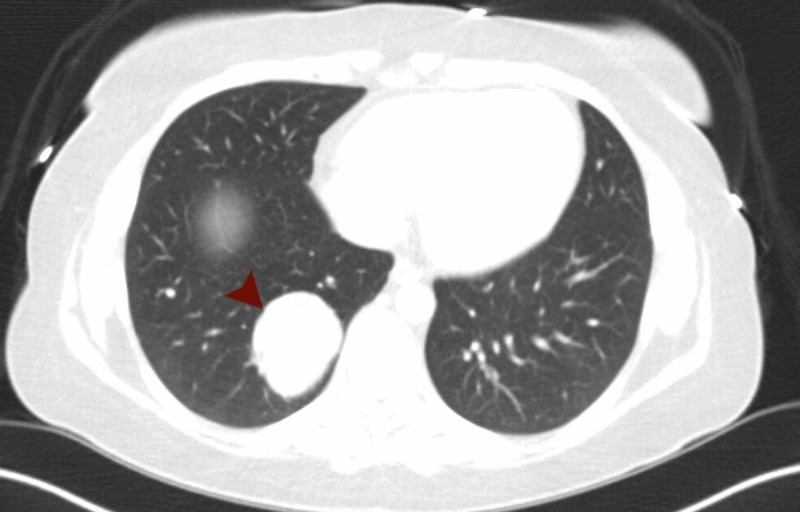
Pulmonary mass (red arrow indicates mass).

The patient’s disease status remained stable for three years when she developed enlargement of one of her gastric masses. Upper endoscopy evaluation revealed an ulcerated bleeding gastric mass and evidence of gastric outlet obstruction. She underwent a distal gastrectomy with Billroth II reconstruction. Pathology was consistent with a GIST. The patient then developed progressive enlargement of her right lower lobe mass two years following gastrectomy. CT chest at time of evaluation revealed a 4.9 x 4.2 cm right lower lobe cystic lesion (Figure 1).

**Figure 2:**
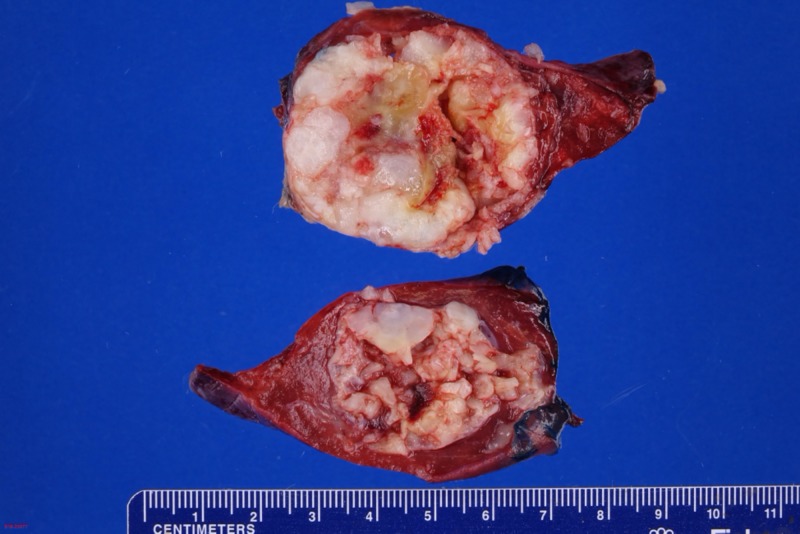
Gross features of bisected pulmonary chondroma: well-circumscribed 4.9 cm lesion composed of varying in size cartilaginous lobules; there is central area of cystic degeneration.

**Figure 3:**
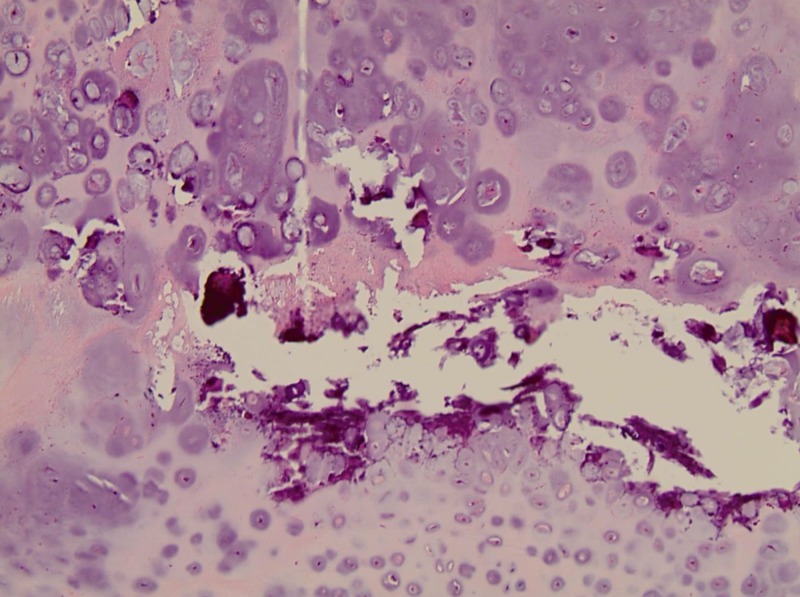
Histologic features of pulmonary chondroma: the entire lesion is composed of mature cartilage with mild cellular pleomorphism; there are foci of microscopic calcification but no other tissues and no entrapment of pulmonary parenchyma (hematoxylin and eosin stain, x200 original magnification).

Following appropriate preoperative evaluation, she underwent an uncomplicated robotic-assisted right lower lobe wedge resection with negative margins. Pathology demonstrated a pulmonary chondroma with three reactive level 9R lymph nodes (Figures 2, 3). A diagnosis of Carney’s triad was made, and she remains on maintenance tyrosine kinase inhibitor with non-progressive disease. 

## Discussion

The disorder of synchronous or metachronous gastric GIST, pulmonary chondroma, and extra-adrenal paraganglioma was first described by Carney et al. in 1977, and thereafter came to be known as the Carney’s triad [1]. Few patients have all three tumors (22%), and therefore the presence of just two of the three tumors is considered a sufficient diagnostic criterion [2].

GIST is the most commonly observed (99%) tumor in the triad, and gastrointestinal bleed or abdominal pain is often the presenting symptom [2]. GISTs most frequently harbor mutations in the proto-oncogene KIT (75%-80% of cases) [3]. This allows for treatment with selective tyrosine kinase inhibitor targeting KIT as in this case. Surgical resection remains the only curative treatment modality; however, recurrence after resection is common, occurring in 46% of patients within 1-36 years after surgery [4]. The stomach is the most common site of occurrence of GIST followed by the small bowel, rectum, and esophagus with up to 50% of patients presenting with metastasis at the time of diagnosis. Metastatic disease is treated with a combination of medical and surgical therapies. The liver and peritoneum are the two most common sites of extra-intestinal metastasis [5]. Metastatic disease to the lung and adrenal has been reported but are uncommon and should prompt further evaluation to rule out Carney’s triad [6].

Pulmonary chondromas are rare benign lung tumors, present in 76% of cases of Carney’s triad, and GIST plus pulmonary chondroma was the most common combination of tumors (53%) [7,8]. The pulmonary tumors are well-differentiated benign cartilaginous lesions that exhibit calcifications (Figures 2, 3). Lymph node pathology shows reactive hyperplasia, as in the present case. These tumors are characteristically asymptomatic and identified radiographically or histologically. They can be single, multiple, and bilateral, without predilection for a specific lobe and can be identified on chest X-ray, enhanced CT scan of the chest, or MRI. On CT scan, they present as round or oval nodules, often measuring between 1.0 and 4.0 cm with mild lobulation, of moderate soft tissue density, inhomogeneous density, with calcification and clear boundaries [8]. The pulmonary lesions can be treated with surgical resection with thoracotomy or through minimally invasive thoracoscopic or robotic approaches [9]. The lesions can also be observed over time without excision; however, the probability of developing symptoms and malignant transformation increases with tumor size [8]. In the case of suspected metastatic GIST tumor with lung tumor, pulmonary wedge resections are helpful in establishing the diagnosis of Carney’s triad in some cases. 

Extra-adrenal gangliomas were present in 47% of Carney’s case series [4,7]. The common locations included carotid body, sympathetic chain, retroperitoneum, and adrenal glands [2]. In a retrospective review, these lesions were characteristically multifocal (22%); 16% were functional pheochromocytoma and 13% were non-functioning adrenocortical tumors [4]. Elevated plasma levels of catecholamines are found in cases of functioning paraganglioma [2]. Surgical resection was curative in most cases (90%), and inoperable tumors were treated with phenoxybenzamine hydrochloride, chemotherapy, radiation, or polyvinyl alcohol particle embolization [4]. 

## Conclusions

Each of the characteristic tumors in Carney’s triad is rare; however patients who develop these lesions in clusters should undergo thorough and ongoing surveillance, particularly if the patient is female, younger than 40 years, and has multifocal tumors. Surgical resection remains the mainstay of therapy and has curative potential. Long tumor-free intervals have been reported prior to recurrence or development of new sites of disease. Lifelong surveillance is recommended. Carney’s triad should be considered in a patient with metastatic GIST and lung tumor. 
